# 99-Case Study of Sporadic Aortic Dissection by Whole Exome Sequencing Indicated Novel Disease-Associated Genes and Variants in Chinese Population

**DOI:** 10.1155/2020/7857043

**Published:** 2020-10-02

**Authors:** Zanxin Wang, Xianmian Zhuang, Bailang Chen, Junmin Wen, Fang Peng, Xiling Liu, Minxin Wei

**Affiliations:** ^1^Department of Cardiac Surgery, Fuwai Hospital Chinese Academy of Medical Sciences, Shenzhen, Guangdong, China; ^2^Department of Cardiac Surgery, Shenzhen Sun Yat-sen Cardiovascular Hospital, Guangdong, China; ^3^Department of Intensive Care, Fuwai Hospital Chinese Academy of Medical Sciences, Shenzhen, Guangdong, China; ^4^Department of Intensive Care, Shenzhen Sun Yat-sen Cardiovascular Hospital, Guangdong, China

## Abstract

**Background:**

In this study, the whole exome sequencing in human aortic dissection, a highly lethal cardiovascular disease, was investigated to explore the aortic dissection-associated genes and variants in Chinese population.

**Methods:**

Whole exome sequencing was performed in 99 cases of aortic dissection. All single nucleotide polymorphisms (SNPs), insertions/deletions (InDels), and copy number variations (CNVs) were filtered to exclude the benign variants. Enrichment analysis and disease-gene correlation analysis were performed.

**Results:**

3425873 SNPs, 685245 InDels, and 1177 CNVs were identified, and aortic dissection-associated SNPs, InDels, and CNVs were collected. After the disease correlation analysis, 20 candidate genes were identified. Part of these genes such as *MYH11*, *FBN1*, and *ACTA2* were consistent with previous studies, while *MLX*, *DAB2IP*, *EP300*, *ZFYVE9*, *PML*, and *PRKCD* were newly identified as candidate aortic dissection-associated genes.

**Conclusion:**

The pathogenic and likely pathogenic variants in most of AD-associated genes (*FBN1*, *MYH11*, *EFEMP2*, *TGFBR2*, *FBN2*, *COL3A1*, and *MYLK*) were identified in our cohort study, and pathogenic CNVs involved in *MYH11*, *COL* family, and *FBN* were also identified which are not detectable by other NGS analysis. The correlation between *MLX*, *DAB2IP*, *EP300*, *ZFYVE9*, *PML*, *PRKCD*, and aortic dissection was identified, and *EP300* may play a key role in AD.

## 1. Introduction

Aortic dissection (AD) is a rare but serious cardiovascular emergency, refers to the blood in aortic lumen that enters the aortic media from the tear of aortic intima, which leads to the separation and expansion along the long axis of the aorta and to form a true and false separation of the aortic wall [1, 2]. By population-based studies, the incidence of AD is approximately 3 cases per 100,000 people annually [3]. Although the incidence of aortic dissection is low, it is very serious. Aortic dissection can quickly lead to death from not enough blood flow to the heart or rupture of the aorta [4]. Meanwhile, aortic dissections are preventable by timely surgical intervention. The identification and clinical screening of at-risk relatives are clinically highly relevant and recommended. Of note, although sporadic cases of AD clearly exist, few studies have evaluated the genetic basis of those patients.

Over the last decade, advances in clinical genetics have led to the identification of disease-associated genes at a rapid pace. Next-generation sequencing (NGS), especially for whole exome sequencing (WES), allows for the rapid analysis of multiple genes in a diagnostic setting at relatively low costs. Especially when surveillance, early detection, and/or treatment provide health benefits for AD patients and their at-risk relatives, identification of an underlying genetic cause for AD is urgent needed [5]. At present, studies on AD genetics have confirmed that *FBN1*, *MYH11*, and *TGFBR1/2* are pathogenic genes, but these genes do not account for 80% of AD cases.

This study is aimed at assessing both a cohort study and the whole exome sequencing in the gene-disease association study. Cohort study will be helpful for genetic diagnosis of AD patients, so as to predict disease prognosis and recurrence risk more accurately and indicate the possible new pathogenic key genes and potential signaling pathways. In our study, 99 AD cases were collected and the overall variants including SNPs, InDels, and CNVs were analyzed. A correlation analysis also was performed by comparing with a Chinese genomics database (Novo zhonghua) to eliminate the genetic background interference, and candidate genes were identified. In addition, 2 GEO databases (GSE52093 and GSE98770) and protein-protein interactions network analysis are performed to analyze the AD-related genes identified in our study. The current study will underline the importance of WES analysis in disease-associated genes and variant researches.

## 2. Methods

### 2.1. Patient Collection

99 patients (94 males and 5 females) with an average age of 47.4 were suspected as sporadic aortic dissection according to chest radiography examinations. Later, they were diagnosed as aortic dissection by spiral CT. Their height, weight, and body mass index (BMI) range are 172.2 ± 8.2 cm, 76.1 ± 10.0 kg, and 25.7 ± 3.4, respectively. 57 of them have a history of smoking, and 82 of them suffered from hypertension. Other complications include diabetes (4 cases), cerebral infarction (3 cases), coronary heart disease (4 cases), renal insufficiency (2 cases), and peptic ulcer (1 case). Venous blood samples were collected from all patients. Candidates with Marfan syndrome, Ehlers-Danlos syndrome, bicuspid aortic valve problems, and previous cardiac surgery were excluded. Candidate samples were collected in Shenzhen Sun Yat-sen Cardiovascular Hospital. These candidates were from all over China, not belonged to one or several specific provinces.

### 2.2. Whole Blood Processing and DNA Isolation

EDTA-anticoagulated blood samples were collected from all candidates referred to the hematology department for routine investigations. Each sample was divided into three equal aliquots of 300 *μ*l whole blood. Until DNA isolation was performed, all blood samples were stored at –80°C.

DNA isolation was performed with the QIAamp blood mini kit (Qiagen, Leusden, Netherlands), the High Pure PCR Template Preparation Kit (Roche Diagnostics GmbH Mannheim, Germany), and the Pure gene DNA isolation Kit (Biozyme, Landgraaf, Netherlands), all according to the manufacturers' protocols. Isolated genomic DNA was quantified spectrophotometrically by measuring absorptions at 260, 280, and 320 nm with the Ultrospec 2000 (Pharmacia Biotech, Roosendaal, Netherlands). Absorption at 320 nm (A^320^) gives information about the presence of e.g., phenolic substances, whereas the ratio of the absorptions at 260 nm versus 280 nm (A^260/280^) provides information about impurities due to proteins or RNA.

### 2.3. Genetic Variation Detection

Whole exome sequencing was performed by Novogene Corporation (Chaoyang District, Beijing, China) in September 2018 on Illumina Hiseq 4000 platform. After a series of quality control tests were performed, SNP was identified by SAMtools, and data was filtered by international common filtering standards in all 99 samples. SNPs were annotated by the ANNOVAR software, which is involved in dbSNP database, 1000-person genome project, and other databases. The location information of variation, types, and conservative prediction were counted. InDels and CNVs were also identified and annotated in the same way.

### 2.4. Advanced Variants Analysis

The results of mutation detection are massive, but the real mutation related to disease is rare. To screen out mutations that are really related to the aortic dissection, we further analyzed and screened the results of mutation detection. We mainly used the existing database, software, and other tools, combined with sample information, and the candidate pathogenic mutation was identified.

The screening process of SNP and InDel is as follows: (1) a mutation whose frequency was higher than 1% in the 1000-person genome data (1000g_all), ESP6500 database (esp6500si_all), and gnomAD data (gnomAD_ALL and gnomAD_EAS) was removed; (2) variation in coding region (exonic) or splicing site (±10 bp) was retained; (3) removal of synonymous SNP mutations that are not highly conserved and not predicted as splicing affected by software; (4) mutations are predicted to be harmful or be splicing affected were retained. All filtered SNPs and InDels were classified into pathogenic, likely pathogenic, benign, and likely benign, and variant uncertain significance (VUS) refers to the evidence from the American Society of Medical Genetics and Genomics (ACMG) [6].

The screened CNVs were also classified, benign CNVs (refer to Database DGV and its derived databases StringentLib, InclusiveLib, and DGV.GoldStandard. July2015) were removed, and malignant CNVs (refer to CNVD) were preserved. AD-associated CNVs were identified from these malignant CNVs.

### 2.5. Enrichment Analysis

In organisms, different genes perform their biological functions by coordinating with each other called pathways. In complex diseases, mutations in multiple genes, which are in the same pathway, lead to pathological phenotype. Significant enrichment analysis was used to identify the metabolic pathways and signal transduction pathways in which mutant genes participated. In this study, Gene Ontology (GO) enrichment analysis and Kyoto Encyclopedia of Genes and Genomes (KEGG) pathway analysis were performed.

### 2.6. Database Correlation Analysis

Novo zhonghua database is a high-depth sequencing genome database contributed by Novogene Corporation. All samples (2827 cases so far) in this database were originated from normal Chinese population. The age of chosen healthy volunteers in this database matched our AD patients. The differences in genetic background due to race specificity will be eliminated by correlation analysis with Novo zhonghua database.

2 GEO (Gene Expression Omnibus) databases (GSE52093 and GSE98770) are used to analyze the candidate genes, in which the samples in GSE52093 and GSE98770 are from tissue, not blood.

### 2.7. Variant-Disease Phenotype Correlation Analysis

DisGeNet database is a database focusing on gene-disease association and mutation-disease association. It contains 561,119 gene-disease association records (GDAs) and 135,588 variant-disease association records (VDAs). Based on the related data in this database, the candidate genes and mutation can be obtained from the disease for subsequent analysis.

We used a precise algorithm in Phenolyzer analysis, combined with sequencing results and a variety of databases, to screen and sort genes, and constructed a correlation map between genes and disease phenotypes. The candidate genes were scored according to their correlation with AD.

### 2.8. Protein-Protein Interactions (PPI) Network Analysis

PPI of differentially expressed genes was based on the STRING database, which known and predicted the protein-protein interactions in the target genes or differentially expressed mRNAs. Then, Cytoscape is used to check the degree of interaction in all candidate genes.

### 2.9. Statistical Evaluation

Data were analyzed with SPSS (IBM Statistics v20, Chicago, IL, USA). Correlations were tested with Spearman's rank correlation (Rs). Raw data was given as median with range and presented graphically as boxplots (showing median and quartiles) with outliers (according to Tukey's criteria) indicated separately. Tests were considered statistically significant at *p* ≤ 0.05.

### 2.10. Ethics Statement

This study was approved by the Research Ethics Committee of Shenzhen Sun Yat-sen Cardiovascular Hospital, and informed consent was obtained from each patient.

## 3. Results

### 3.1. AD-Associated SNPs and InDels Filtered

There were 3,425,873 SNPs and 685,245 InDels identified from 99 AD patient samples. In further variants analysis, more than 99% of SNPs and InDels were excluded. 28,223 SNPs and 3,645 InDels, which were harmful based on our algorithm, were retained. In those filtered SNPs and InDels, 294 were pathogenic and 515 were likely pathogenic refer to the guideline of ACMG, and 547 genes that SNPs and InDels located in were involved. AD-associated genes, *FBN1*, *MYH11*, *EFEMP2*, *TGFBR2*, *FBN2*, *COL3A1*, and *MYLK*, were identified in 11 patients. Two pathogenic variants were identified in patient IDs 13 and 20. 12 of these variants were SNPs, and only one base insertion was identified in patient ID 98 (Table 1).

### 3.2. AD-Associated CNVs Identified

In total, 1177 CNVs were identified in this study, in which 239 were deletion and 938 were duplication. 1031 CNVs are located in CDS region (Table 2). 845 CNVs were filtered, and 32 of them were high deleterious which were indicated as a malignant risk in database CNVD but not presented in benign CNV database (DGV). Other CNVs were probably deleterious which were indicated as a malignant risk in database CNVD and also were presented in benign CNV database (DGV).

14 duplications were involved in AD-associated genes including *MYH11*, *COL* family, and *FBN* (Table 3). 11 of these CNVs were presented in patient ID 74. Another 377 CNVs were also identified in this sample, and all of them were duplication.

### 3.3. Enrichment Analysis

**Figure 1:**
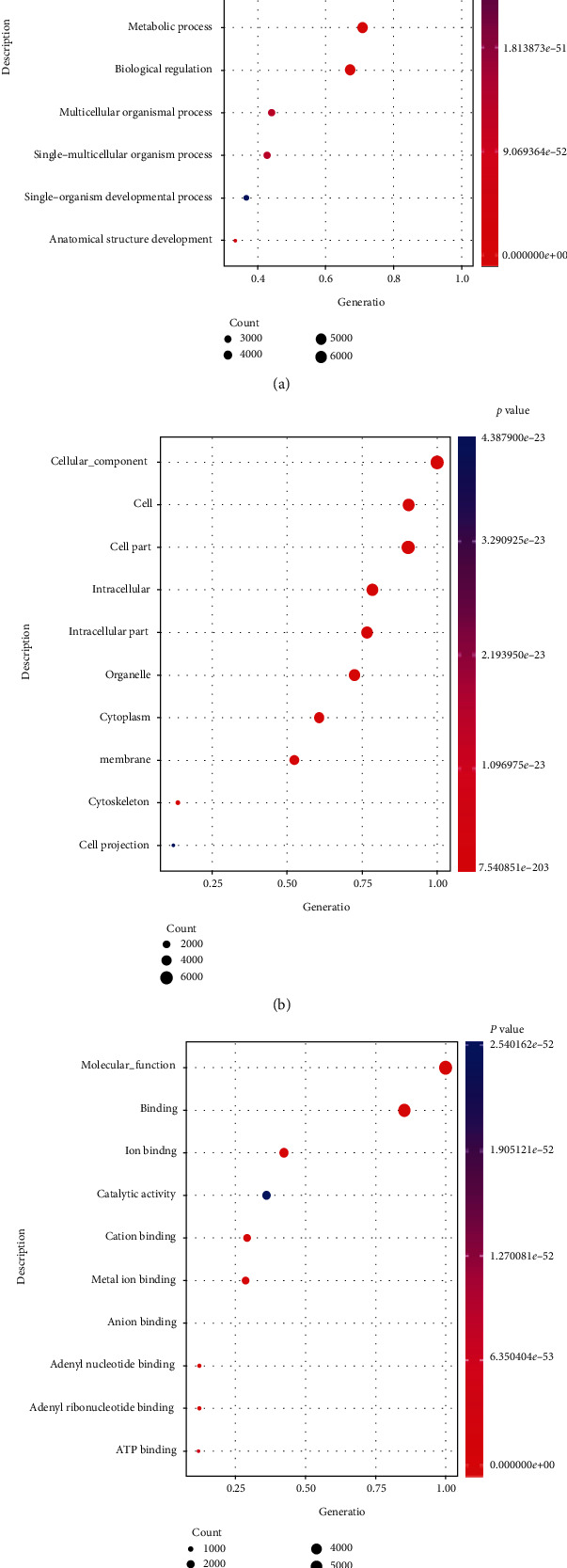
The top 10 GO terms enriched in different expression genes: (a) biological process (BP), (b) cellular component (CC), and (c) molecular function (MF).

In GO enrichment analysis, biological process, cellular process, single-organism process, single-organism cellular process, metabolic process, biological regulation, multicellular organismal process, single-multicellular organism process, single-organism developmental process, and anatomical structure development in biology process category were enriched (Figure 1(a)).

Most genes were enriched in terms of cellular component, cell, cell part, intracellular, intracellular part, organelle, cytoplasm, membrane, cytoskeleton, and cell projection in cell component category (Figure 1(b)).

And molecular function, binding, ion binding, catalytic activity, cation binding, metal ion binding, anion binding, adenyl nucleotide binding, adenyl ribonucleotide binding, and ATP binding enriched the most genes in molecular function category (Figure 1(c)).

**Figure 2:**
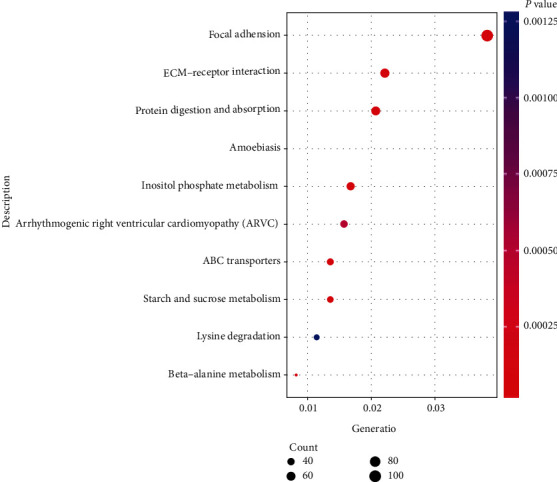
The top 10 GO terms enriched in different expression genes.

In KEGG pathways, the most enriched pathway was focal adhesion. Extracellular matrix- (ECM-) receptor interaction, protein digestion and absorption, amoebiasis, inositol phosphate metabolism, arrhythmogenic right ventricular cardiomyopathy (ARVC), ABC transporters, starch and sucrose metabolism, lysine degradation, and beta-alanine metabolism were also enriched (Figure 2).

AD-associated genes, such as *COL3A1* family, were enriched in protein digestion and absorption and amoebiasis pathway. Other COL proteins were enriched in focal adhesion, ECM-receptor interaction, protein digestion, and absorption pathway. *ACTA2* and *MYH11* were enriched in vascular smooth muscle contraction pathway (hsa04270) whose *p* value was 0.015.

### 3.4. Novo Zhonghua Database Correlation Analysis

By comparing with 2827 cases in Novo zhonghua database, 7374 genes were identified. There were 14 AD-associated genes that were different from Novo zhonghua database (Table 4). The mutations of ACTA2 in our 99 cases were much more than that of Novo zhonghua. In all 99 cases (198 gene loci), 4 of them were mutation. In 2827 healthy cases (5654 gene loci), there were 5 mutation identified. The mutation frequency in our AD patients was much higher than that in Novo zhonghua database. For example, the mutation frequency of *FBN1* and *SLC2A10* was also much higher in 99 AD patients than that in Novo zhonghua database. The difference was significant (*p* value < 0.1).

### 3.5. 20 Candidate Genes Were Identified, and 6 Genes Were Newly Identified to Be Associated with AD

**Figure 3:**
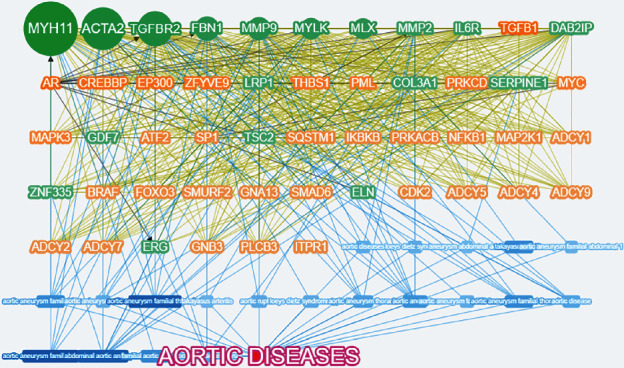
The associated genes of AD by Phenolyzer database.

Combining the sequencing results and various databases, 18 AD-associated genes (green gene) and 32 related genes (orange gene) were sequenced, and the association map between genes and disease phenotypes was constructed (Figure 3). All these genes were associated with AD and other aortic diseases, such as aneurysm. An aortic aneurysm is a localized, abnormal, weak spot on a blood vessel wall that causes an outward bulging, likened to a bubble or balloon, and is thought to be an increased risk for AD.

**Figure 4:**
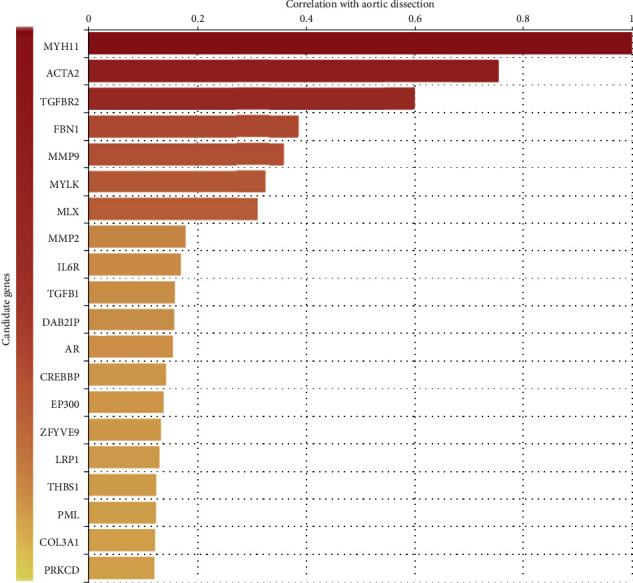
The top 20 highest correlation genes with AD.

Using our algorithm and correlation analysis results, 20 genes were collected which were most relevant to AD (Figure 4). The most relevant gene was *MYH11*, which product is a major contractile protein, converting chemical energy into mechanical energy through the hydrolysis of ATP. *MYH11* is affecting the C-terminal coiled-coil region of the smooth muscle myosin heavy chain. Mutations in *MYH11* cause a marked aortic stiffness, which is associated with thoracic aortic aneurysm/aortic dissection.

There were previous studies that show evidence for the relationship with AD, such as *ACTA2*, *TGFBR2*, *FBN1*, *MMP2*, *MMP9*, *MYLK*, *TGFB1*, *COL3A1*, *IL6R*, *AR*, *CREBBP*, *LRP1*, and *THBS1. MLX*, *DAB2IP*, *EP300*, *ZFYVE9*, *PML*, and *PRKCD* were newly identified to be associated with AD based on current literature retrieval and analysis. Further researches will be needed to verify the mechanism that these genes involved in AD.

### 3.6. *EP300*, *FBN1*, and Other 7 Candidate Genes Were Differentially Expressed in GSE52093

**Figure 5:**
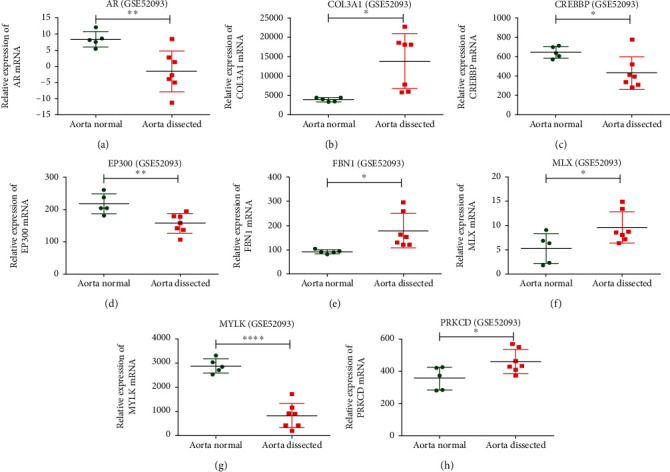
The differentially expressed candidate genes in GSE52093 database.

In GSE52093, 4,212 DEGs for AD were identified and 17 of 20 candidate genes were analyzed. *AR*, *COL3A1*, *CREBBP*, *EP300*, *FBN1*, *MLX*, *MYLK*, and *PRKCD* were differentially expressed between AD tissue and vascular tissue of healthy people (Figure 5). For newly identified genes, *EP300* and *MLX* were downregulated in AD tissue, and *PRKCD* was upregulated.

In GSE98770, 663 DEGs for AD were identified and 11 of 20 candidate genes were analyzed. All candidate genes involved in this database were not differentially expressed.

### 3.7. EP300 Plays a Key Role in AD

**Figure 6:**
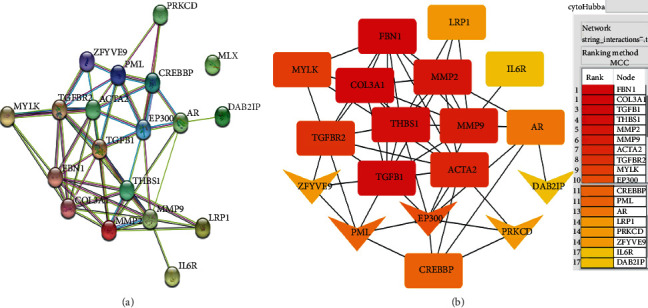
PPI correlation analysis for candidate gene.

The PPI network shows that there are several core genes in candidate gene interaction network (Figure 6(a)). *TGFB1* is the core gene in the network and interacts with the most candidate genes. In addition, there are several subcore genes, such as *TGFBR2*, *FBN1*, *ACTA2*, *THBS1*, and *EP300*.

The interaction among candidate genes in the Cytoscape software shows similar mode (Figure 6(b)). *TGFB1*, *FBN1*, and *COL3A1* (rectangle nodes) were top 3 in network string_interactions-.tsv ranked by the MCC method. And *EP300* (arrow node) interacted with *TGFB1*, *ACTA2*, *THBS1*, *AR*, *CREBBP*, and other AD-related genes and was the highest-ranked gene which was newly identified.

## 4. Discussion

Using whole exome sequencing technique, we systematically identified the variants and genes that were associated with AD. Not only those AD-associated genes in previous studies were identified, novel AD-associated genes were also involved in our study. Importantly, we found that cohort analysis combined with next-generation sequencing is a new way to identify the gene-disease gene for rare disease, such as AD.

In the past, monogenic syndromes and some extreme phenotypes were used for the identification of disease-associated genes. The syndromes that affect blood vessel wall strength will probably related with AD, such as Marfan syndrome and Ehlers-Danlos syndrome, a bicuspid aortic valve, and previous heart surgery [7, 8]. Heterozygous mutation of *COL3A1* on chromosome 2q32 can cause vascular Ehlers-Danlos syndrome (EDS-VASC). Schwarze et al. identified 30 *COL3A1* splice point mutations and three small deletions (the splicing sequence and some exons deleted) in 33 unrelated patients with type IV EDS [9].

*MYH11* mutation will harden the vascular wall which greatly increases the risk of dissection of blood vessels [10, 11]. In patients with *MYH11* variants, the effect of intronic variants on splicing was classified into pathogenic or nonpathogenic variants in aortic aneurysms, so as to *ACTA2* variants [12]. In patient 6 of our study, we identified a SNP (rs267606902) which was classified to be likely pathogenic mutations by ACMG. But in ClinVar database, this SNP was pathogenic. Five benign variants were found in *MYH11* in our study.

*Fibrillin*-*1* (*FBN*) is an extracellular matrix glycoprotein that serves as a structural component of calcium-binding microfibrils. Diseases associated with *FBN1* were thought to be Marfan lipodystrophy syndrome, and part of the mutation were identified as c.8175_8182del8bp, p.Arg2726Glufs∗9 in exon 64 of the *FBN1* gene [13]. In another study, *FBN1* in 687 of nonsyndromic AD was sequencing and 27 of them were identified the variants (27/687, 3.9%) which were significantly high in the sporadic nonsyndromic AD cohort [14]. In this study, 3 pathogenic variants (patient IDs: 13, 45, and 89) and 2 likely pathogenic variants (patient IDs: 3 and 35) were identified. The frequency of mutation was 5%, slightly higher than the previous study and the novo zhonghua database (1.7%-3.5%). There were other biomarkers for AD, such as *sST2* (ST2 is an interleukin-1 receptor family member with transmembrane (ST2L) and sST2, a soluble truncated form of ST2L). In our study, the variants of *sST2* were not identified [15].

There were also several novel-associated genes identified. These genes include *MLX*, *DAB2IP*, *EP300*, *ZFYVE9*, *PML*, and *PRKCD*, and they were more relevant to aortic dissection than other genes. But in previous study, no AD-associated function and pathway were found to be involved in these genes. *MLX* belongs to the family of basic helix-loop-helix leucine zipper (bHLH-Zip) transcription factors. A recent genome-wide association study (GWAS) in Han Chinese population has revealed that several genes including *MLX* confer susceptibility to Takayasu arteritis (TA) [16]. Aortic dissection is a rare complication of Takayasu arteritis [17]. So *MLX* may also affect the development of AD. But further mechanism of this gene needs to be studied. *DAB2IP* is a Ras GTPase-activating protein (GAP) and functions as a scaffold protein implicated in the regulation of a large spectrum of both general and specialized signaling pathways. The variants have been associated with aneurysms of the abdominal aorta (AAA) in genome-wide association studies [18]. However, there was no replication in multiple cohorts. Our study in AD, which is related with aneurysms, may provide support for the previous AAA study. *ZFYVE9* recruits *SMAD2* to the TGF-*β* receptor and was differentially expressed and contributed to aortic dilatation in turner syndrome [19]. *PML* contributes to interferon *α*-mediated inhibition of angiogenesis and alters the angiostatic activity [20]. Aortic dissection is more frequently in antiangiogenic therapy process [21]. But there was no direct evidence for *PML* in this process as well as *EP300* and *PRKCD* [22–24].

*EP300* may play a key role in AD because it interacts with 7 AD-related genes in our study. *EP300* is a cellular p300 transcriptional coactivator protein and functions as histone acetyltransferase. *EP300* also works as a coactivator of *HIF1A* (hypoxia-inducible factor 1 alpha) and interacts with the latter by the TAZ-type 1 domain [25]. *HIF1A* and *EP300* play roles in the stimulation of *VEGF* together. Considering the critical role of *VEGF* signaling in the homeostasis of the cardiovascular system, *EP300* may participate in the AD pathological process via *VEGF*.

There are several limitations in our study. The main problem is that the number of samples in our study is limited by the low incidence of aortic dissection. More samples cannot be collected in time. Still, we identified most of AD-associated genes which are consistent with previous studies. However, for our novel AD-associated genes, the evident we provided in this study is insufficient. Second, further studies will be carried out to verify the repeatability and reliability of these novel AD-associated genes. NGS sequencing has many false-positive and false-negative results, and the underlying pathophysiologic mechanism of novel AD-associated genes needs to be understood.

In conclusion, pathogenic and likely pathogenic variants in most of AD-associated genes (*FBN1*, *MYH11*, *EFEMP2*, *TGFBR2*, *FBN2*, *COL3A1*, and *MYLK*) were identified in our cohort study, and pathogenic CNVs involved in *MYH11*, *COL* family, and *FBN* were also identified, which are not detectable by other NGS analysis. Comparing with Chinese genomics database, the mutation frequency of *ACTA2* and other two genes in our 99 cases was significantly higher after eliminating racial genetic background. Our study is the correlation analysis that shows *MLX*, *DAB2IP*, *EP300*, *ZFYVE9*, *PML*, and *PRKCD* were AD-associated genes, and *EP300* may play a key role in AD. Our study underlines the importance of cohort analysis combined with whole exome sequencing in the disease-related gene exploration.

## Figures and Tables

**Table 1:** AD-associated SNPs and InDels identified in 99 patients.

Patient ID	Avsnp	REF	ALT	GeneName	Func	ACMG	ClinVar
3	rs727503057	G	A	FBN1	Exonic	Likely pathogenic	Pathogenic
6	rs267606902	C	T	MYH11	Exonic	Likely pathogenic	Pathogenic
13	rs532989312	G	A	EFEMP2	Exonic	Likely pathogenic	
13		T	C	FBN1	Exonic	Pathogenic	
17	rs779131465	A	G	TGFBR2	Splicing	Pathogenic	
20		C	T	FBN2	Splicing	Pathogenic	
20	rs104893809	C	T	TGFBR2	Exonic	Likely pathogenic	Pathogenic
35		A	G	FBN1	Splicing	Likely pathogenic	
36		G	A	TGFBR2	Splicing	Pathogenic	
38		T	C	COL3A1	Exonic	Pathogenic	
45	rs193922219	C	T	FBN1	Splicing	Pathogenic	Pathogenic
89		C	A	FBN1	Exonic	Pathogenic	
98		C	CAGAA	MYLK	Exonic	Likely pathogenic	

Patient ID: patient number; Avsnp: the dbSNP ID (https://www.ncbi.nlm.nih.gov/snp/); REF: the base in reference genome; ALT: the base in our study; GeneName: gene symbol; Func: the location of the variant; ACMG: the ACMG pathological rate; ClinVar: the ClinVar database pathological rate.

**Table 2:** CNV identification.

Var type	Total number	CDS	Splicing	UTR5	UTR3	Intron	Upstream	Downstream	ncRNA	Intergenic	Unknown
Total	1177	1031	0	0	0	2	1	0	77	66	0
Del	239	197	0	0	0	0	0	0	18	24	0
Dup	938	834	0	0	0	2	1	0	59	42	0

Dup: increased copies of CNV; Del: reduced number of copies; CDS: number of CNVs in coding sequence; splicing: number of CNVs in splicing region (±10 bp of splicing site); UTR5: number of CNVs in 5′UTR; UTR3: number of CNVs in 3′UTR; intron: number of CNVs in the intron region; upstream: number of CNV in 1 Kb region upstream of transcription start site; downstream: number of CNV in downstream 1 Kb region of transcription termination site; ncRNA: number of CNV in noncoding RNA region; intergenic: number of CNV in the intergenic region; unknown: unknown functional loci due to the imperfection of the annotation database of gene structure.

**Table 3:** AD-associated CNVs.

Patient ID	Priority	Chr	GeneName	Func	Size	CNVType
18	H-	16	NDE1, KIAA0430, MPV17L, ABCC6, ABCC1, C16orf45, MIR6506, FOPNL, MYH11, MIR484	Exonic	806641	Dup
25	H-	16	FOPNL, NDE1, ABCC1, KIAA0430, NOMO3, ABCC6, MIR6506, MPV17L, C16orf45, MIR484, MYH11	Exonic	856226	Dup
28	P-	17	SHISA6, MYHAS, SCO1, ADPRM, MAGOH2P, MYH3, LINC00675, PIRT, MYH2, TMEM220-AS1, TMEM220	Exonic	697923	Dup
74	H-	7	COL1A2, CASD1	Exonic	89899	Dup
74	P	12	COL2A1	Exonic	4682	Dup
74	P-	16	MYH11, NDE1	Exonic	21043	Dup
74	P-	17	MYH8, MYHAS	Exonic	6040	Dup
74	P-	19	FBN3	Exonic	8402	Dup
74	P-	19	MYH14	Exonic	13571	Dup
74	P-	2	LOC654841, COL4A3	Exonic	11221	Dup
74	P-	4	COL25A1	Exonic	16573	Dup
74	P-	4	COL25A1	Exonic	37877	Dup
74	P-	5	COL23A1, CLK4	Exonic	317238	Dup
74	P-	8	COL14A1	Exonic	42568	Dup

Patient ID: patient number; priority: CNV pathological rate; H (high): evident in the malignant database (CNVD) and no record in the benign database (DGV); P (possibly deleterious): no record in the benign and malignant database; GeneName: gene symbol; Func: the location of the variant; size: the length of CNV; CNVType: the type of CNV (duplication or deletion).

**Table 4:** AD-associated genes in Novo zhonghua database correlation analysis.

Gene	Alt_case	Ref_case	Alt_control	Ref_control	*p* value
ACTA2	4	194	5	5649	0.00014
FBN1	8	190	99	5555	0.02789
SLC2A10	3	195	30	5624	0.09926
ELN	3	195	46	5608	0.2297
MYH11	5	193	87	5567	0.24114
TGFBR2	2	196	27	5627	0.25712
COL5A1	1	197	105	5549	0.26919
NOTCH1	2	196	130	5524	0.32839
TGFB2	1	197	11	5643	0.33863
COL3A1	2	196	36	5618	0.37014
FBN2	6	192	124	5530	0.4541
PLOD1	2	196	54	5600	0.71451
COL5A2	2	196	79	5575	1
MYLK	2	196	60	5594	1

Gene: gene name; Alt_case: number of mutated alleles in our 99 cases; Ref_case: number of nonmutated alleles in 99 cases; Alt_control: number of mutated alleles in control 2827 Novo zhonghua cases; Ref_control: number of nonmutated alleles in control 2827 Novo zhonghua cases; *p* value: *p* value obtained by Fisher's test between our cases and cases in Novo zhonghua database.

## Data Availability

All data and protocols used for this study are either included in the article (or in its supporting files) or are available upon request.
